# Genomic Selection for Wheat Blast in a Diversity Panel, Breeding Panel and Full-Sibs Panel

**DOI:** 10.3389/fpls.2021.745379

**Published:** 2022-01-07

**Authors:** Philomin Juliana, Xinyao He, Felix Marza, Rabiul Islam, Babul Anwar, Jesse Poland, Sandesh Shrestha, Gyanendra P. Singh, Aakash Chawade, Arun K. Joshi, Ravi P. Singh, Pawan K. Singh

**Affiliations:** ^1^Borlaug Institute for South Asia (BISA), Ludhiana, India; ^2^International Maize and Wheat Improvement Center (CIMMYT), Mexico, Mexico; ^3^Instituto Nacional de Innovación Agropecuaria y Forestal (INIAF), La Paz, Bolivia; ^4^Bangladesh Wheat and Maize Research Institute (BWMRI), Dinajpur, Bangladesh; ^5^Department of Plant Pathology, Wheat Genetics Resource Center, Kansas State University, Manhattan, KS, United States; ^6^Indian Council of Agricultural Research (ICAR)-Indian Institute of Wheat and Barley Research, Karnal, India; ^7^Department of Plant Breeding, Swedish University of Agricultural Sciences, Alnarp, Sweden; ^8^CIMMYT-India, New Delhi, India

**Keywords:** wheat, blast disease, genomic selection (GS), marker-assisted selection, pedigree selection, genotyping-by sequencing, *Magnaporthe oryzae*

## Abstract

Wheat blast is an emerging threat to wheat production, due to its recent migration to South Asia and Sub-Saharan Africa. Because genomic selection (GS) has emerged as a promising breeding strategy, the key objective of this study was to evaluate it for wheat blast phenotyped at precision phenotyping platforms in Quirusillas (Bolivia), Okinawa (Bolivia) and Jashore (Bangladesh) using three panels: (i) a diversity panel comprising 172 diverse spring wheat genotypes, (ii) a breeding panel comprising 248 elite breeding lines, and (iii) a full-sibs panel comprising 298 full-sibs. We evaluated two genomic prediction models (the genomic best linear unbiased prediction or GBLUP model and the Bayes B model) and compared the genomic prediction accuracies with accuracies from a fixed effects model (with selected blast-associated markers as fixed effects), a GBLUP + fixed effects model and a pedigree relationships-based model (ABLUP). On average, across all the panels and environments analyzed, the GBLUP + fixed effects model (0.63 ± 0.13) and the fixed effects model (0.62 ± 0.13) gave the highest prediction accuracies, followed by the Bayes B (0.59 ± 0.11), GBLUP (0.55 ± 0.1), and ABLUP (0.48 ± 0.06) models. The high prediction accuracies from the fixed effects model resulted from the markers tagging the 2NS translocation that had a large effect on blast in all the panels. This implies that in environments where the 2NS translocation-based blast resistance is effective, genotyping one to few markers tagging the translocation is sufficient to predict the blast response and genome-wide markers may not be needed. We also observed that marker-assisted selection (MAS) based on a few blast-associated markers outperformed GS as it selected the highest mean percentage (88.5%) of lines also selected by phenotypic selection and discarded the highest mean percentage of lines (91.8%) also discarded by phenotypic selection, across all panels. In conclusion, while this study demonstrates that MAS might be a powerful strategy to select for the 2NS translocation-based blast resistance, we emphasize that further efforts to use genomic tools to identify non-2NS translocation-based blast resistance are critical.

## Introduction

An emerging threat to wheat production that has the potential to cause substantial yield losses is the disease blast (Kohli et al., 2011; Islam et al., 2016; Chowdhury et al., 2017; Cruz and Valent, 2017; Sadat and Choi, 2017; Singh et al., 2021), caused by the fungus *Magnaporthe oryzae* pathotype *Triticum* Catt. (MoT) (anamorph *Pyricularia oryzae* Cavara) (Couch and Kohn, 2002; Tosa and Chuma, 2014; Zhang et al., 2016). The disease primarily affects the spikes which become partially or fully bleached, resulting in inferior quality of grains which are small, shriveled and have low test weight (Goulart et al., 2007; Urashima et al., 2009; Cruz and Valent, 2017). First identified in 1985 in Brazil (Igarashi, 1986), the disease spread to the major Brazilian wheat growing areas (Goulart et al., 1990; Igarashi, 1990; Picinini and Fernandes, 1990; Dos Anjos et al., 1996), and then moved to Bolivia, Paraguay and Argentina in 1996, 2002, and 2007, respectively (Barea and Toledo, 1996; Viedma and Morel, 2002; Cabrera and Gutiérrez, 2007; Perelló et al., 2015).

The first intercontinental jump of the MoT pathogen from South America to Asia was reported in 2016, when there was a blast outbreak in Bangladesh most likely caused by the South American lineage of MoT via wheat importation (Islam et al., 2016; Malaker et al., 2016; Ceresini et al., 2018). In addition, the warm and humid climate at heading time during that year was also a significant driver of the epidemic, as both high temperatures (between 25 and 30°C) and long wetting periods favor blast development (Cardoso et al., 2008; Islam et al., 2019). Another major intercontinental jump of the MoT pathogen to Africa was recently reported, when blast was observed in the Muchinga province of Zambia during the 2017–2018 rainy season (Tembo et al., 2020). Furthermore, about seven million hectares of wheat growing regions in India, Pakistan and Bangladesh and some states in the United States (Louisiana, Mississippi and Florida) were identified to be vulnerable to blast outbreaks, given their similar favorable environmental conditions (Cruz et al., 2016a; Mottaleb et al., 2018; Valent et al., 2021), indicating that further spread of the disease is possible.

Wheat blast management approaches like the use of fungicides, planting time alteration and discontinuation of wheat cultivation in disease-prone regions by declaring a wheat holiday have only been partly successful in combating the disease (Mottaleb et al., 2019b; Roy et al., 2021). This is because of limitations such as inefficient control with fungicides when the disease pressure is high, inability of poor farmers to afford fungicides, development of resistance to some fungicide classes in MoT populations and challenges of finding the appropriate profitable alternative wheat land use (Urashima et al., 2009; Kohli et al., 2011; Castroagudín et al., 2015; Cruz et al., 2015, 2019; Coelho et al., 2016; Cruz and Valent, 2017; Mottaleb et al., 2019a,b). Hence, the most sustainable, cost-effective and farmer-friendly approach to wheat blast control is developing and deploying blast resistant wheat varieties (Cruz and Valent, 2017).

Genetic resistance to wheat blast is known to follow the gene-for-gene interaction model in the seedling stage (Takabayashi et al., 2002), while field resistance is also known to be quantitative (Goddard et al., 2020; He et al., 2021). Among the five reported wheat blast resistance genes including *Rmg2, Rmg3, Rmg7, Rmg8*, and *RmgGR119*, only the genes *Rmg8* and *RmgGR119* are known to be effective against several recent MoT isolates (Zhan et al., 2008; Anh et al., 2015, 2018; Tagle et al., 2015; Cruz and Valent, 2017; Wang S. et al., 2018). Besides these genes, the 2NS translocation from the wild species, *Aegilops ventricosa* has been reported to confer a consistent and strong effect on blast resistance in several studies, although the resistance is sometimes background dependent and partial (Cruz et al., 2016b; Juliana et al., 2019, 2020a; He et al., 2020, 2021; Ferreira et al., 2021; Wu et al., 2021).

Breeding for wheat blast resistant genotypes first involves screening to find resistant germplasm and then identifying resistance genes. However, wheat breeding programs globally are constrained in their ability to screen a large number of lines for blast resistance, as phenotyping can only be done in the blast hot-spot locations and there is a limitation to the number of lines that can be handled, unless their phenotyping capacity is expanded. While this poses a huge challenge to accelerate development of blast resistant wheat varieties, it is an excellent case for the application of genomic selection (GS), an approach that was advocated to change the role of phenotyping in breeding (Heffner et al., 2009). Using GS, breeders can eliminate phenotyping and select genotypes based on their genomic-estimated breeding values (GEBVs) for traits, that are obtained from genome-wide markers (Meuwissen et al., 2001). In GS, a “training population” that includes lines that have been genotyped and phenotyped for the trait of interest is used to train prediction models that are then used to obtain the GEBVs of individuals (also known as “selection candidates” or “testing population”) that have been only genotyped. While GS has proved to be effective in predicting quantitative disease resistance (Ornella et al., 2012; Rutkoski et al., 2014; Juliana et al., 2019), it also has the potential to increase the accuracy of selection, reduce cycle time and cost, thereby leading to an increase in gain from selection (Heffner et al., 2010; Voss-Fels et al., 2019).

Given the potential of GS for wheat blast, the key objective of this study was to evaluate it in the following panels, assuming that a subset or half of them were phenotyped: (a) **Diversity panel** comprising diverse spring wheat lines and varieties that were developed over several years by the International Maize and Wheat Improvement Centre (CIMMYT) and South Asia partners, which is useful to understand if GS can be applied to select for blast resistance in unrelated lines or any set of existing historic germplasm. (b) **Breeding panel** comprising elite lines from CIMMYT’s international nurseries, which is useful to understand if GS can be applied to select advanced breeding lines for blast resistance. (c) **Full-sibs panel** comprising progenies from a cross between a resistant and a susceptible blast parent, which is useful to understand if selection for blast is effective within families, i.e., among sister lines in biparental populations. The other main objectives of this study were to:

(i) compare genomic prediction accuracies from the genomic best linear unbiased prediction (GBLUP) model that utilizes the genomic relationships between lines (de los Campos et al., 2013; Habier et al., 2013) and the Bayes B model that utilizes the estimated marker effects (Meuwissen et al., 2001) to generate GEBVs.
(ii) compare genomic prediction accuracies from both the genomic prediction models (GBLUP and Bayes B) with prediction accuracies from a fixed effects model, where a genome-wide association analysis for blast is first done in the training set, followed by selection of the best model (when adding a marker to the model no longer increases the prediction accuracy) and use of the selected marker(s) to estimate the breeding values, referred to as the estimated breeding values (EBVs).
(iii) compare prediction accuracies from the GBLUP model and the fixed effects model to the accuracies from the combined GBLUP and the fixed effects model (GBLUP + fixed effects).
(iv) compare genomic prediction accuracies with pedigree-based prediction accuracies, where pedigree-based relationships between the lines is used to obtain the EBVs, in a pedigree (additive)-best linear unbiased prediction model (ABLUP).
(v) compare selections made from the blast phenotypes (phenotypic selection, PS) with selections using the EBVs from the different models to understand what percentage of lines that are selected and discarded by PS, overlap with the breeding values-based selections.
(vi) test the hypothesis that GS would perform better than the selections based on EBVs from a fixed-effects model (which can be considered similar to marker-assisted selection, MAS) and the pedigree relationships-based model (pedigree selection).
(vii) compare prediction accuracies in subsets of lines with and without the 2NS translocation in the three panels using the GBLUP, Bayes B, fixed effects, GBLUP + fixed effects and ABLUP models.

## Materials and Methods

### Panels, Blast Evaluation Sites, Crop Cycles, and Planting Time

#### Diversity Panel

The diversity panel comprised 172 diverse spring wheat genotypes including lines developed by CIMMYT and varieties released in South Asia (India, Bangladesh, and Nepal), some of which were directly introduced from CIMMYT. The diversity panel was phenotyped for blast in two planting dates that were about 14 days apart, indicated as first planting (FP) and second planting (SP) in the following blast precision phenotyping platforms and crop cycles:

(i) Quirusillas, Bolivia (18°20′S 63°57′W) during the 2017–2018 and 2018–2019 crop cycles (December to April) in two different planting dates and the datasets are referred to by the site followed by the harvest year and planting time as: Quirusillas 2018 FP, Quirusillas 2018 SP, Quirusillas 2019 FP and Quirusillas 2019 SP.
(ii) Okinawa, Bolivia (17°13′S 62°53′W) during the 2018 crop cycle (May to September) in two planting dates and the datasets are referred to as Okinawa 2018 FP and Okinawa 2018 SP.
(iii) Jashore, Bangladesh (23°10′N 89°10′E) during the 2017–2018 crop cycle (December to April) in two different planting dates and the datasets are referred to as Jashore 2018 FP and Jashore 2018 SP.

#### Breeding Panel

The breeding panel comprised 248 lines from CIMMYT’s international nurseries that included subsets of lines from the 50th International Bread Wheat Screening Nursery (IBWSN, 119 lines) and the 35th Semi-Arid Wheat Screening Nursery (SAWSN, 129 lines). The IBWSNs and SAWSNs comprise advanced breeding lines developed by CIMMYT’s global wheat program using the selected bulk-breeding scheme that are targeted to the irrigated and drought-prone target environments, respectively and are CIMMYT’s primary vehicles of germplasm dissemination globally (Rajaram et al., 1993; van Ginkel and Rajaram, 1993). From the set of 269 lines from the 50th IBWSN and 265 lines from the 35th SAWSN (Juliana et al., 2020a), subsets of lines were chosen after filtering out a large number of lines that had across-environment blast best linear unbiased estimates (BLUEs) of 0, and only some of those lines were retained to avoid a large number of lines with a blast index of 0 in the training and prediction populations. Similarly, only the environments where more than half the entries did not have a blast index of 0 were chosen. The selected environments where the breeding panel was phenotyped for blast included:

(i) Quirusillas during the 2017–2018 crop cycle (December to April) in the FP date and in the 2018–2019 crop cycle in two different planting dates and the datasets are referred to as: Quirusillas 2018 FP, Quirusillas 2019 FP and Quirusillas 2019 SP.
(ii) Okinawa during the 2018 crop cycle (May to September) where only the second planting was chosen (Okinawa 2018 SP), due to the high number of resistant lines in the FP.

#### Full-Sibs Panel

The full-sibs panel comprised 298 full-sibs or F_2:7_ recombinant inbred lines that were obtained by single seed descent from a cross between a resistant female parent Caninde#1 (with the 2NS translocation) and a susceptible male parent Alondra (without the 2NS translocation), as described in He et al. (2020). The full-sibs panel was phenotyped for blast in two planting dates in the following sites and crop cycles:

(i) Quirusillas during the 2017–2018 and 2018–2019 crop cycles (December to April) in two different planting dates and the datasets are referred to as: Quirusillas 2018 FP, Quirusillas 2018 SP, Quirusillas 2019 FP and Quirusillas 2019 SP.
(ii) Okinawa during the 2018 and 2019 crop cycles (May to September) in two planting dates and the datasets are referred to as Okinawa 2018 FP, Okinawa 2018 SP, Okinawa 2019 FP and Okinawa 2019 SP.
(iii) Jashore during the 2017–2018 and 2018–2019 crop cycles (December to April) in two planting dates and the datasets are referred to as Jashore 2018 FP, Jashore 2018 SP, Jashore 2019 FP and Jashore 2019 SP.

#### Blast Phenotyping—Field Experimental Design, Inoculation, Evaluation, and Analyses

In all the three sites, the lines were planted in double rows each of 1-m length with 20-cm spacing in between them. Blast inoculation in Quirusillas and Okinawa was done using isolates QUI1505, QUI1601, QUI1612, OKI1503, and OKI1704 and in Jashore it was done using isolates BHO17001, MEH17003, GOP17001.2, RAJ17001, CHU16001.3, and JES16001, all of which were collected locally and exhibited high pathogenesis. Inoculum was prepared according to He et al. (2020) by culturing the MoT isolates on oatmeal agar medium. Inoculum concentration was adjusted to 80,000 spores/mL and applied using a backpack sprayer at anthesis, followed by a second inoculation 2 days later, in all the environments.

Disease development after inoculation was favored using a misting system that was set up to provide 10 min of misting each hour, between 8 a.m. and 7 p.m. in the Bolivian sites and between 9 a.m. to 5 p.m. in Jashore. In addition to the panel lines, local checks were also planted and evaluated for blast, which included resistant check Urubo and susceptible check Atlax in Bolivia and resistant check BARI Gom 33 (Hossain et al., 2019) and susceptible check BARI Gom 26 in Jashore. Evaluation of wheat blast was done 21 days post the first inoculation on 10 spikes marked at anthesis, where the total number of spikelets and those infected were counted. Wheat blast index was obtained using the formula: index = incidence (proportion of spikes that had blast infection) × severity (average percentage of infected spikelets).

The BLUEs for blast in each of the panels were calculated using the ASREML statistical package (Gilmour, 1997) using the following mixed model:


(1) $$y_{ij}=\mu +g_{i}+e_{j}+\varepsilon_{ij}$$

where *y*_*ij*_ is the observed blast index, μ is the overall mean, *g*_*i*_is the fixed effect of the genotype, *e_j_* is the random effect of the environment (site-year-planting time) that was independent and identically distributed (IID) ($e_{j}∼N\left(0,\sigma_{e}^{2}\right)$), and ε_*ij*_ is the residual with IID $\left(\varepsilon_{ij}∼N\left(0,\sigma_{\varepsilon }^{2}\right)\right)$. Analysis of the blast indices in the different panels and environments was done and the mean, standard deviation, median, minimum and maximum blast indices were obtained in all the datasets. Visualization of all the results in this study was done using the “R” package “ggplot2” (Wickham, 2009). The narrow-sense heritabilities for blast across the different environments in each panel were obtained using the formula:


(2) $$h^{2}=\frac{\sigma_{A}^{2}}{\sigma_{A}^{2}+\sigma_{\varepsilon }^{2}}$$

where $\sigma_{A}^{2}$ was the additive genetic variance among the lines calculated using markers and $\sigma_{\varepsilon }^{2}$ is the error variance. The heritabilities, genetic and error variances were obtained using the average information-restricted maximum likelihood algorithm (Gilmour et al., 1995) in the “R” package “heritability” (Kruijer et al., 2015).

#### Genotyping

The diversity panel was genotyped for genome-wide markers using the Illumina Infinium 15K BeadChip (TraitGenetics, Germany) and four sequence tagged site (STS) markers associated with the *Yr17* gene in the 2NS translocation namely: *Ventriup* (Helguera et al., 2003), *WGGB156* and *WGGB159* (Wang Y. et al., 2018) and *cslVrgal3* (Seah et al., 2001; He et al., 2021). The breeding panel was genotyped for genome-wide markers using the genotyping-by-sequencing (GBS) platform (Poland et al., 2012) and the TASSEL (Trait Analysis by aSSociation Evolution and Linkage) version 5 GBS pipeline (Glaubitz et al., 2014) was used to call the marker polymorphisms. Marker polymorphisms discovery, alignment to the reference genome assembly (RefSeq v1.0) of Chinese Spring (IWGSC, 2018) and tag filtering were done as described in Juliana et al. (2020a). The full-sibs panel was genotyped for genome-wide markers using the DArTseq platform (Genetic Analysis Service for Agriculture, CIMMYT, Mexico), the four STS markers mentioned above and also another marker *IWB11136* tagging the 2NS translocation (Xue et al., 2018). The genome-wide markers in each panel and STS markers were filtered for those with less than 60% missing data, greater than 10% minor allele frequency and less than 10% heterozygosity resulting in 13,427 markers in the diversity panel, 8,072 markers in the breeding panel and 2,489 markers in the full-sibs panel. Marker imputation in all the panels was done using the linkage disequilibrium k-nearest neighbor genotype imputation method (Money et al., 2015) in TASSEL version 5 (Bradbury et al., 2007).

#### Blast Prediction

Blast prediction in all the panels was done using a twofold cross-validation approach, where each of the panels was divided into two random folds and one-half of the lines was used to predict the breeding values of the other half of the lines for blast within each panel. We have only evaluated twofold cross-validations, because across the panels, 15.8–62.5% of the lines had a blast index of zero and dividing them into smaller folds might result in some random folds having most of the lines with a blast index of zero. The sampling of the random folds was iterated 10 times, the prediction accuracy was calculated as the Pearson’s correlation between the observed blast index values and the breeding values in each iteration and the mean prediction accuracy across the 10 iterations was obtained for each of the datasets in the different panels using the following models:

(i) **Fixed effects model**

For the fixed effects model implemented in “R,” a stepwise least-squares approach was used which involved the following steps:

• Identification of markers significantly associated with blast in the training set using a genome-wide association analysis and calculation of marker *p*-values.
• Ranking of markers according to their *p*-values.
• Marker selection from the ranked markers was done with the following stepwise regression model:

(3) $$\text{y}=1_{n}\mu +{\text{X}}_{i}\beta_{i}\ldots {\text{X}}_{j}\beta_{j}+\varepsilon$$

where *y* was the blast phenotype, μ was the mean, β_*i*_and β_*j*_ were the effects of the *i*th and *j*th marker, and **X_i_** and **X_j_** were the *i*th and *j*th marker’s genotype matrix and ε was the error term. Here, for each iteration *i* through *j*, we added a marker to the model, starting from the marker that had the lowest *p*-value. We then calculated the twofold cross validation accuracy within the training set after each iteration and selected the model that had *j*-1 markers, when the accuracy*_*j*_*_–1_ was greater than the accuracy*_*j*_*.

• Estimation of marker effects was done from the selected markers, and the effects were subsequently used for obtaining the EBVs of lines in the testing populations for blast resistance.

(ii) **Genomic-best linear unbiased prediction (GBLUP)**

The GBLUP model was fitted using the “R” package “rrBLUP” (Endelman, 2011) and can be represented by the following mixed model:


(4) $$\text{y}=\mu 1+{\text{Z}}_{g}\text{u}+\varepsilon$$

where ***y*** was the vector of blast indices, μ was the mean, *u* represented the additive genetic effects, **Z** was the design matrix for the random effects and **ε** was the error term. The joint distribution of **u** (the vector of additive genetic effects) was assumed to be multivariate normal i.e., $MN\left(0,\text{G}\sigma_{g}^{2}\right)$, where **G** was the marker-based genomic relationship matrix calculated using the method of VanRaden (2008) [**G** = **ZZ**′/*p*, where **Z** was the centered and standardized marker matrix and *p* was the number of markers] and σ^2^_*g*_ was the genetic variance]. The joint distribution of **ε** (error term) was also assumed to be multivariate normal i.e., *MN* (**0**, **I**σ^2^_*e*_), where **I** was the identity matrix and σ^2^_*e*_ was the residual variance.

(iii) **Genomic-best linear unbiased prediction and fixed effects (GBLUP + fixed effects)**

In the GBLUP + fixed effects model, in addition to modeling the markers as random effects in the GBLUP model, some loci were also modeled as fixed effects and the model can be represented as:


(5) $$\text{y}=1_{n}\mu +{\text{X}}_{i}\beta_{i}\ldots {\text{X}}_{j}\beta_{j}+{\text{Z}}_{g}\text{u}+\varepsilon$$

where the terms are the same as described in (3) and (4).

(iv) **Bayes B**

In the Bayes B model (Meuwissen et al., 2001), a mixture distribution prior is used, where some marker effects are assumed to be zero with probability, π (the markers linked to regions of the genome that have no effect on the trait and hence zero effect), and some marker effects are assumed to be drawn from a scaled-*t* distribution with probability, 1-π (the markers linked to regions of the genome that have an effect on the trait). The Bayes B model was fitted in the “R” package “BGLR” (Pérez and de los Campos, 2014), using the default prior parameters and 10,000 iterations, while the first 1,000 iterations were discarded as burn-in.

(v) **Pedigree-best linear unbiased prediction (ABLUP)**

The ABLUP was a modified version of the GBLUP that was also implemented in the “R” package “BGLR,” where the marker-based genomic relationship matrix was replaced by the pedigree-based relationship matrix, that was calculated from the coefficient of parentage and the pedigree tracing back to five generations.

To compare the prediction accuracies obtained from the different models and to test if they were significantly different from each other, we performed paired-*t*-tests using the ‘‘JMP’’ statistical software^1^ and obtained the mean differences between the prediction accuracies from the different model pairs in each panel. We also obtained the *p-*values to test their significance at a threshold of 0.005 for three alternate hypotheses: the mean prediction accuracy of one model is significantly greater or less than the other model (two-tailed *t*-test), the mean prediction accuracy of one model is significantly greater than the other model (one-tailed *t*-test) and the mean prediction accuracy of one model is significantly lesser than the other model (one-tailed *t*-test).

#### Comparison of Genomic Selection With Marker-Assisted Selection and Pedigree-Based Selection

The BLUEs dataset in all the panels was used to select the most resistant blast lines using the phenotypes (PS) and compared to the following selections made using the EBVs for blast obtained from different models: (i) MAS using the EBVs obtained from the fixed effects model (ii) GS using the GEBVs obtained from the GBLUP (GS GBLUP) and Bayes B (GS Bayes B) models (iii) GS + MAS using the GEBVs obtained from the GBLUP + fixed effects model (iv) pedigree selection using the EBVs obtained from the ABLUP model. For PS, we selected the lines with blast indices less than 10 in the BLUEs dataset for all the panels and an equal number of lines were selected using the EBVs obtained from the different models.

#### Blast Prediction in Subsets of Lines With and Without the 2NS Translocation

Subsets of lines with and without the 2NS translocation were obtained using consensus data from the STS markers tagging the 2NS translocation in the diversity and full-sibs panels and using all the 2AS markers significantly associated with blast in the fixed effects model in the breeding panel. The lines where the presence or absence of the 2NS translocation could not be determined using all the markers (because of missing data or contrasting information from different markers) were excluded from predictions. Within the subsets of lines with and without the 2NS translocation, blast prediction was done using twofold cross-validations with the fixed effects, GBLUP, Bayes B, GBLUP + fixed effects and ABLUP models. The mean prediction accuracies obtained from the subsets with and without the 2NS translocation in each of the panels were compared using paired-*t*-tests.

## Results

### Diversity Panel

#### Statistical Analysis of Blast Indices in the Diversity Panel

Statistical analysis of blast indices in the diversity panel (Supplementary Data 1 and Table 1) indicated that the mean blast indices were relatively higher in the Quirusillas 2019 FP (38.5 ± 35.1), Quirusillas 2018 FP (32 ± 25.5) and Okinawa 2018 SP (31.4 ± 22.9) datasets. The maximum blast index in the individual diversity panel datasets ranged between 48 and 100. We also observed that 23.3% (Jashore 2018 SP) to 43% (Quirusillas 2018 SP) of the lines in the different environments had a blast index of zero. The phenotypic correlations between the blast indices in the two plantings were high in Quirusillas 2019 (0.7), while they were moderate in Okinawa 2018 (0.58), Quirusillas 2018 (0.56), and Jashore 2018 (0.46). Across the sites of blast evaluation, we observed low to moderate correlations between the blast indices in Jashore and the Bolivian sites (ranged between 0.27 and 0.53), while moderate to high correlations (ranged between 0.47 and 0.67) were observed between the blast indices in Okinawa and Quirusillas. The narrow-sense heritability of blast across all the environments in the diversity panel was 0.38 ($\sigma_{A}^{2}$ = 190.1 and $\sigma_{\varepsilon }^{2}$ = 308.2).

**TABLE 1:** Statistical analysis of blast indices in the diversity panel with 172 lines.

Dataset	Mean	Standard deviation	Median	Minimum	Maximum
Quirusillas 2018 FP	32.0	25.5	35.9	0	90.7
Quirusillas 2018 SP	22.3	24.1	11.0	0	77.0
Quirusillas 2019 FP	38.5	35.1	36.9	0	100.0
Quirusillas 2019 SP	29.9	27.4	27.9	0	98.2
Okinawa 2018 FP	21.8	20.5	18.3	0	76.4
Okinawa 2018 SP	31.4	22.9	38.7	0	72.7
Jashore 2018 FP	11.2	12.3	8.9	0	48.0
Jashore 2018 SP	18.5	17.5	14.4	0	74.1
BLUEs	25.7	18.2	28.3	0	61.3

*FP, First planting; SP, Second planting; BLUEs, Best linear unbiased estimates.*

#### Prediction Accuracies for Blast in the Diversity Panel

**FIGURE 1:**
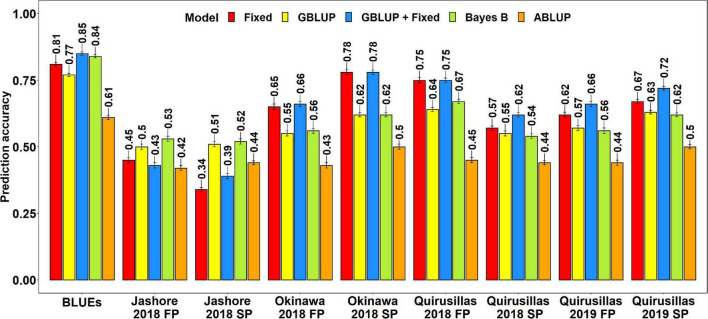
Twofold cross validation prediction accuracies for blast response in the diversity panel (172 lines) using the fixed effects (Fixed), genomic best linear unbiased prediction (GBLUP), GBLUP and fixed effects (GBLUP + Fixed), Bayes B, and pedigree best linear unbiased prediction (ABLUP) models. FP refers to the first planting, SP refers to the second planting and BLUEs refer to the best linear unbiased estimates of blast indices across the different environments.

The mean prediction accuracies for blast across the different environments for all the lines in the diversity panel were: (i) 0.63 ± 0.14 using the GBLUP + fixed effects model (ii) 0.60 ± 0.15 using the fixed effects model (iii) 0.58 ± 0.05 using the Bayes B model (iv) 0.57 ± 0.05 using the GBLUP model and (v) 0.45 ± 0.03 using the ABLUP model (Figure 1). In the fixed effects model, one or two markers on chromosome 2AS (Supplementary Table 1) that were selected by association analysis and stepwise regression were used as fixed effects in the different datasets (except the Jashore 2018 FP and SP datasets). This included markers Tdurum_contig29983_490 (259,187 bps, 0 cM), Kukri_c22599_114 (397,565 bps, 0 cM), Tdurum_contig11802_864 (2,478,927 bps, 0 cM), Ventriup (3,965,255 bps, 0 cM), wsnp_Ku_c33374_42877546 (4,789,998 bps, 2.9 cM), Kukri_c31776_1621 (7,550,063 bps, 8.9 cM), AX-94629608 (14,327,985 bps, 8.9 cM) and AX-94684111 (27,276,097 bps, 9.8cM), that were located between 259,187 and 27,276,097 bps on the Refseq v1.0 (IWGSC, 2018) and between 0 and 9.8 cM on the Popseq map (Chapman et al., 2015).

With all the models, the blast BLUEs had the highest prediction accuracies (0.61–0.85) in the diversity panel, that were 34.2–45.5% higher than the mean prediction accuracies of the individual environments. Considering all the models, we observed that the mean prediction accuracy was the highest in Okinawa 2018 SP (0.66 ± 0.12) and the lowest in Jashore 2018 SP (0.44 ± 0.08) dataset. We observed that the mean differences in prediction accuracies were not significant in the two-tailed *t*-tests at a threshold of 0.005 for the following model pairs:

(i) GBLUP + fixed effects and Bayes B: Mean difference = 0.04, *p-*value = 0.21
(ii) GBLUP + fixed effects and GBLUP: Mean difference = 0.06, *p-*value = 0.09
(iii) GBLUP + fixed effects and fixed effects: Mean difference = 0.02, *p-*value = 0.03
(iv) Bayes B and GBLUP: Mean difference = 0.01, *p-*value = 0.17
(v) Fixed effects and Bayes B: Mean difference = 0.02, *p-*value = 0.57
(vi) Fixed effects and GBLUP: Mean difference = 0.03, *p-*value = 0.33

However, the prediction accuracies from all models were significantly higher than the prediction accuracies from the ABLUP model at a threshold of 0.005 (*p-*values for the one-sided *t*-test ranged from 4.03 × 10^–6^ to 2 × 10^–3^) and the mean differences in prediction accuracies between the ABLUP and other models ranged from 0.12 to 0.18.

#### Phenotypic Selection vs. Estimated Breeding Values Based Selection for Blast in the Diversity Panel

**FIGURE 2:**
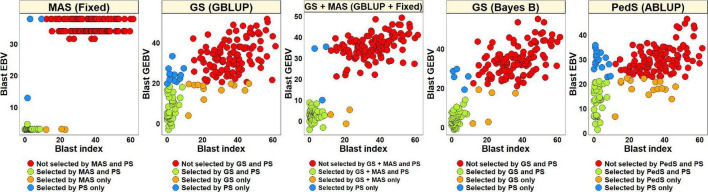
Comparison of phenotypic selection (PS) of the best linear unbiased estimates of blast indices across environments with: (i) marker assisted selection (MAS) using the estimated breeding values (EBVs) obtained from the fixed effects model (Fixed) (ii) genomic selection (GS) using the genomic estimated breeding values (GEBVs) obtained from the genomic best-linear unbiased prediction (GBLUP) and Bayes B models (iii) GS + MAS using the GEBVs obtained from the GBLUP and fixed effects (GBLUP + Fixed) model and (iv) pedigree selection (PedS) using the EBVs obtained from the pedigree best linear unbiased prediction (ABLUP) model in the diversity panel comprising 172 lines.

For PS, we selected 52 lines (30.2%) with blast indices less than 10 in the BLUEs dataset and an equal number of lines using the EBVs from different models (Figure 2). Considering the GS + MAS and MAS, we observed that among the 52 lines selected by PS, 94.2% were also selected by these two selection methods. Similarly, 90.4 and 76.9% lines were selected by GS, using the GEBVs obtained from the Bayes B and GBLUP models, respectively. Among the 120 lines that were discarded by PS, 97.5, 97.5, 95.8, and 90% were also discarded by GS + MAS, MAS, GS Bayes B and GS GBLUP, respectively. However, considering pedigree selection, we observed that 69.2% lines that were selected by PS were also selected by pedigree selection, while 86.7% lines that were not selected by PS were also not selected by pedigree selection.

#### Blast Distribution and Prediction Accuracies in Subsets of Lines With and Without the 2NS Translocation in the Diversity Panel

**FIGURE 3:**
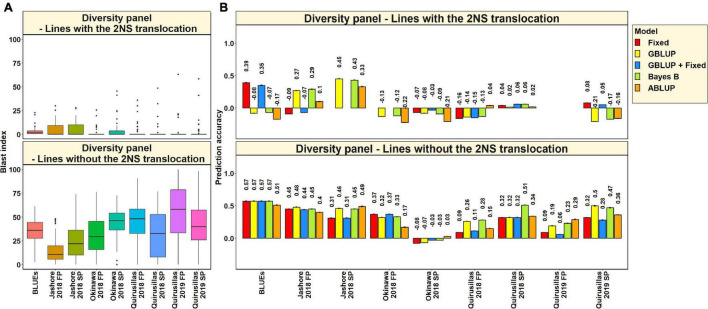
**(A)** Boxplots showing the wheat blast indices in 53 lines with the 2NS translocation in the diversity panel and 119 lines without the 2NS translocation in the diversity panel. **(B)** Two-fold cross validation prediction accuracies for blast response in 53 lines with the 2NS translocation and 119 lines without the 2NS translocation in the diversity panel using the fixed effects (Fixed), genomic best linear unbiased prediction (GBLUP), GBLUP and fixed effects (GBLUP + Fixed), Bayes B, and pedigree best linear unbiased prediction (ABLUP) models. The prediction accuracies are missing for some environments and models in the subset of lines with the 2NS translocation, where several lines had a blast index of zero. In **(A,B)**, FP refers to the first planting, SP refers to the second planting and BLUEs refer to the best linear unbiased estimates of blast indices across the different environments.

In the 53 diversity panel lines with the 2NS translocation, we observed that the mean blast index ranged between 1.5 ± 4.8 and 4.9 ± 7.3 in the different environments (Figure 3A). Similarly, in the 119 diversity panel lines without the 2NS translocation, the mean blast index ranged between 14.3 ± 12.9 and 54.6 ± 29.8 in the different environments. The mean prediction accuracies for blast across the different environments for the lines with the 2NS translocation in the diversity panel ranged between 0.04 ± 0.17 using the GBLUP + fixed effects model and −0.03 ± 0.19 using the ABLUP model (Figure 3B). Similarly, for the lines without the 2NS translocation in the diversity panel, the mean prediction accuracies ranged between 0.36 ± 0.18 using the Bayes B model and 0.27 ± 0.19 using the GBLUP + fixed effects model. The prediction accuracies could not be obtained for some environments and models in the subset of lines with the 2NS translocation, as several lines had a blast index of zero. The markers used as fixed effects in the different datasets for the lines with and without the 2NS translocation in the diversity panel are given in Supplementary Tables 2, 3, respectively.

We observed that the mean prediction accuracy across all the environments and models was significantly higher in the subset of lines without the 2NS translocation compared to the subset of lines with the 2NS translocation (mean difference = 0.29, *p-*value = 1.2 × 10^–4^). In the diversity panel lines where the 2NS translocation was present, the mean prediction accuracy across all the models was the highest in Jashore 2018 SP (0.4 ± 0.06) and the lowest in Okinawa 2018 FP (−0.16 ± 0.06). In the diversity panel lines where the 2NS translocation was absent, we observed that the blast BLUEs had the highest mean prediction accuracy (0.56 ± 0.03) across all the models, and Okinawa 2018 SP had the lowest mean prediction accuracy (−0.04 ± 0.04).

### Breeding Panel

#### Statistical Analysis of Blast Indices in the Breeding Panel

Statistical analysis of blast indices in the breeding panel (Table 2) indicated that the mean blast indices was the highest in Quirusillas 2019 FP (14.6 ± 27.5) and lowest in Quirusillas 2018 FP (10.2 ± 19.2). While the maximum blast indices ranged between 68.6 and 100 in the different datasets, 48% (Quirusillas 2019 SP) to 62.5% (Quirusillas 2018 FP) of the lines in the different environments had a blast index of zero. The phenotypic correlation between the blast indices in the Quirusillas 2019 FP and SP was very high (0.82). The Okinawa 2018 SP dataset also had high correlations (ranged between 0.70 and 0.75) with the Quirusillas blast evaluations. The narrow-sense heritability of blast across all the environments in the breeding panel was 0.65 $\sigma_{A}^{2}$ 318 and $\sigma_{\varepsilon }^{2}$ = 168).

**TABLE 2:** Statistical analysis of blast indices in the breeding panel with 248 lines.

Dataset	Mean	Standard deviation	Median	Minimum	Maximum
Okinawa 2018 SP	10.6	18.1	0	68.6	0
Quirusillas 2018 FP	10.2	19.2	0	87.2	0
Quirusillas 2019 FP	14.6	27.5	0	100	0
Quirusillas 2019 SP	14.1	24.7	1	94.1	0
BLUEs	12.4	20.1	2.3	77.9	0

*FP, First planting; SP, Second planting; BLUEs, Best linear unbiased estimates.*

#### Prediction Accuracies for Blast in the Breeding Panel

**FIGURE 4:**
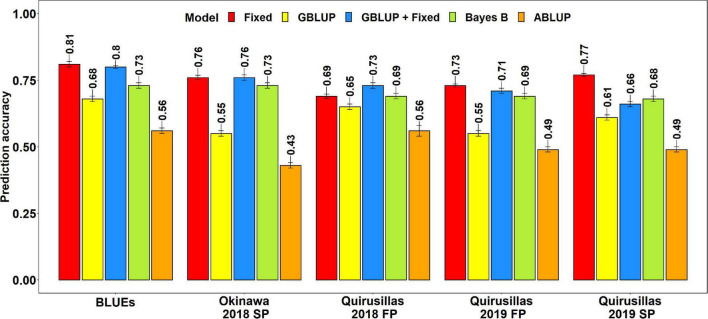
Twofold cross validation prediction accuracies for blast response in the breeding panel (248 lines) using the fixed effects (Fixed), genomic best linear unbiased prediction (GBLUP), GBLUP and fixed effects (GBLUP + Fixed), Bayes B, and pedigree best linear unbiased prediction (ABLUP) models. FP refers to the first planting, SP refers to the second planting and BLUEs refer to the best linear unbiased estimates of blast indices across the different environments.

The mean prediction accuracies for blast in the breeding panel using different models were: (i) 0.75 ± 0.04 using the fixed effects model (ii) 0.73 ± 0.05 using the GBLUP + fixed effects model (iii) 0.70 ± 0.02 using Bayes B model (iv) 0.61 ± 0.06 using the GBLUP model and (v) 0.51 ± 0.06 using the ABLUP model (Figure 4). In the fixed effects model, one to four selected markers on chromosome 2AS (Supplementary Table 4) were used as fixed effects in the different datasets of the breeding panel. This included markers 2A_718152 (718,152 bps, 0 cM), 2A_1686041 (1,686,041 bps, 0 cM), 2A_1872142 (1,872,142 bps, 0 cM) and 2A_2367215 (2,367,215 bps, 0 cM), that were located between 718,152 and 2,367,215 bps on the Refseq v1.0 (IWGSC, 2018) and at 0 cM on the Popseq map (Chapman et al., 2015).

The highest mean prediction accuracy with the different models in the breeding panel was observed in the blast BLUEs dataset (0.56-0.81). However, unlike in the diversity panel, the accuracies in the blast BLUEs dataset from each model were only 4.7–15.3% higher than the mean prediction accuracies of the individual environments. Across all the models, we observed that the mean prediction accuracy was the highest in the Quirusillas 2018 FP (0.66 ± 0.06) dataset and the lowest in Quirusillas 2019 FP (0.63 ± 0.11) dataset.

The tests for the significance of the mean differences between the prediction accuracies obtained from the different models indicated that they were not significant in the two-tailed *t*-tests at a threshold of 0.005 for the following model pairs:

(i) GBLUP + fixed effects and Bayes B: Mean difference = 0.03, *p-*value = 0.13
(ii) GBLUP + fixed effects and fixed effects: Mean difference = 0.02, *p-*value = 0.01
(iii) Bayes B and fixed effects: Mean difference = 0.05, *p-*value = 0.02.

However, the Bayes B, GBLUP + fixed effects and fixed effects models had significantly higher prediction accuracies compared to the GBLUP model, with the mean differences ranging between 0.10 and 0.14 and the *p-*values ranging between 7.6 × 10^–5^ and 2.4 × 10^–3^. Similarly, all the marker-based models had significantly higher prediction accuracies compared to the ABLUP model, with the mean differences ranging between 0.10 and 0.25 and the *p-*values ranging between 3.6 × 10^–6^ and 5.3 × 10^–4^.

#### Phenotypic Selection vs. Estimated Breeding Values Based Selection for Blast in the Breeding Panel

**FIGURE 5:**
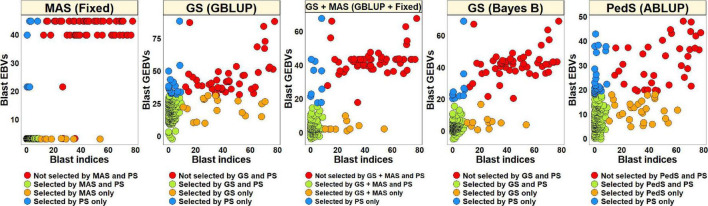
Comparison of phenotypic selection (PS) of the best linear unbiased estimates of blast indices across environments with: (i) marker assisted selection (MAS) using the estimated breeding values (EBVs) obtained from the fixed effects model (fixed) (ii) genomic selection (GS) using the genomic estimated breeding values (GEBVs) obtained from the genomic best-linear unbiased prediction (GBLUP) and Bayes B models (iii) GS + MAS using the GEBVs obtained from the GBLUP and fixed effects (GBLUP + Fixed) model and (iv) pedigree selection (PedS) using the EBVs obtained from the pedigree best linear unbiased prediction (ABLUP) model in the breeding panel comprising 248 lines.

To compare PS and EBVs-based selection for blast resistance using the BLUEs dataset in the breeding panel, we selected 185 lines (74.6%) with blast indices less than 10 and an equal number of lines using the EBVs (Figure 5). The highest percentage of overlap with PS was obtained using the EBVs from the fixed effects model, where 95.7% lines were selected by both MAS and PS, while 87.3% of the lines were not selected by both. Selection from the GEBVs obtained from the GBLUP + fixed effects, Bayes B and GBLUP models resulted in selection of 94.6, 94, and 90.3% lines, respectively, that were also selected by PS and discarding of 84.1, 82.5, and 71.4% lines, respectively, that were also discarded by PS. However, in pedigree selection using the EBVs from the ABLUP model, 85.4% lines overlapped with the lines selected by PS and 57.1% lines overlapped with the lines discarded by PS.

#### Blast Distribution and Prediction Accuracies in Subsets of Lines With and Without the 2NS Translocation in the Breeding Panel

**FIGURE 6:**
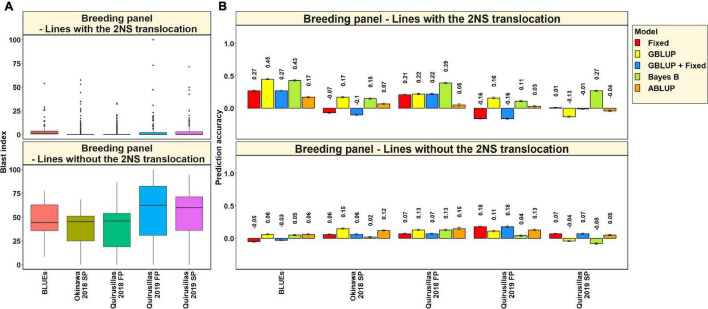
**(A)** Boxplots showing the wheat blast indices in 185 lines with the 2NS translocation in the breeding panel and 47 lines without the 2NS translocation in the breeding panel. **(B)** Two-fold cross validation prediction accuracies for blast response in 185 lines with the 2NS translocation and 47 lines without the 2NS translocation in the breeding panel using the fixed effects (Fixed), genomic best linear unbiased prediction (GBLUP), GBLUP and fixed effects (GBLUP + Fixed), Bayes B, and pedigree best linear unbiased prediction (ABLUP) models. In **(A,B)**, FP refers to the first planting, SP refers to the second planting and BLUEs refer to the best linear unbiased estimates of blast indices across the different environments.

In the 185 lines with the 2NS translocation in the breeding panel, we observed that the mean blast index ranged between 2.3 ± 5.4 and 3.7 ± 8.9 in the different environments (Figure 6A). In the 47 lines without the 2NS translocation in the breeding panel, the mean blast index ranged between 38.2 ± 18.7 and 56.8 ± 30.3 in the different environments. The mean prediction accuracies for blast across the different environments for the lines with the 2NS translocation in the breeding panel ranged between 0.27 ± 0.14 using the Bayes B model and 0.04 ± 0.19 using the GBLUP + fixed effects model (Figure 6B). Similarly, for the lines without the 2NS translocation in the breeding panel, the mean prediction accuracies ranged between 0.10 ± 0.04 using the ABLUP model and 0.03 ± 0.08 using the Bayes B model. The markers used as fixed effects in the different datasets for the lines with and without the 2NS translocation in the breeding panel are given in Supplementary Tables 5, 6, respectively.

We observed that in the subsets of lines with and without the 2NS translocation, the mean prediction accuracy was not significantly different (mean difference = 0.05, *p-*value = 0.25). In the breeding panel lines with the 2NS translocation, we observed that the blast BLUEs had the highest mean prediction accuracy (0.32 ± 0.12) across all the models, and Quirusillas 2019 FP had the lowest mean prediction accuracy (−0.004 ± 0.15). In breeding panel lines without the 2NS translocation, the mean prediction accuracy across all the models was the highest in Quirusillas 2019 FP (0.13 ± 0.06) and the lowest in Quirusillas 2019 SP (0.01 ± 0.07).

### Caninde#1 × Alondra Full-Sibs Panel

#### Statistical Analysis of Blast Indices in the Caninde#1 × Alondra Full-Sibs Panel

In the Caninde#1 × Alondra full-sibs panel (Table 3), we observed that the mean blast indices were the highest in the Okinawa 2019 FP (55.7 ± 41.8) dataset. While the maximum blast index was 100 in nine out of the 12 datasets, we also observed that 15.8% (Jashore 2019 FP) to 42.3% (Quirusillas 2018 FP) of the lines in the different datasets had a blast index of zero. Across the different planting times, we observed moderate to high correlations between the blast indices ranging between 0.87 in Okinawa 2019 and 0.58 in Jashore 2018. Considering the different sites of blast evaluation, we observed moderate correlations between the blast indices in Jashore and the Bolivian sites (ranged between 0.39 and 0.69), while high to very high correlations (ranged between 0.58 and 0.82) were observed between the blast indices in Okinawa and Quirusillas. The narrow-sense heritability of blast across all the environments in the full-sibs panel was 0.55 ($\sigma_{A}^{2}$ = 633.7 and $\sigma_{\varepsilon }^{2}$ = 520.9).

**TABLE 3:** Statistical analysis of blast indices in the Caninde#1 × Alondra full-sibs panel with 298 lines.

Dataset	Mean	Standard deviation	Median	Minimum	Maximum
Quirusillas 2018 FP	19.4	21.3	13.4	0	90.1
Quirusillas 2018 SP	32.4	28.4	37.9	0	100
Quirusillas 2019 FP	41.6	36.9	43.1	0	100
Quirusillas 2019 SP	43.6	38.4	48.1	0	100
Jashore 2018 FP	29.1	28.1	24.5	0	100
Jashore 2018 SP	28.5	24.8	24.7	0	100
Jashore 2019 FP	32.3	25.3	30.8	0	100
Jashore 2019 SP	45.8	40.0	39.5	0	100
Okinawa 2018 FP	34.8	28.6	42.6	0	92.9
Okinawa 2018 SP	28.0	25.8	29.3	0	96.0
Okinawa 2019 FP	55.7	41.8	70.3	0	100
Okinawa 2019 SP	46.5	39.3	49.5	0	100
BLUE	36.5	25.6	45.5	0	90.1

*FP, First planting; SP, Second planting; BLUEs, Best linear unbiased estimates.*

#### Prediction Accuracies for Blast in the Caninde#1 × Alondra Full-Sibs Panel

**FIGURE 7:**
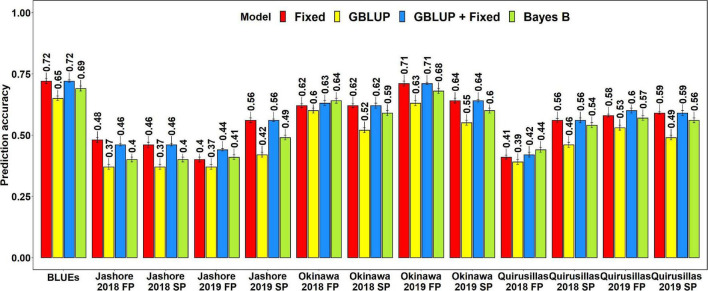
Twofold cross validation prediction accuracies for blast response in the full-sibs panel (298 lines) using the fixed effects (Fixed), genomic best linear unbiased prediction (GBLUP), GBLUP and fixed effects (GBLUP + Fixed) and Bayes B models. FP refers to the first planting, SP refers to the second planting and BLUEs refer to the best linear unbiased estimates of blast indices across the different environments.

The mean prediction accuracies for blast in the Caninde#1 × Alondra population using different models were: (i) 0.57 ± 0.10 using the fixed effects model (ii) 0.57 ± 0.10 using the GBLUP + fixed effects model (iii) 0.54 ± 0.10 using Bayes B model and (iv) 0.49 ± 0.10 using the GBLUP model (Figure 7). In the fixed effects model, one to three selected markers on chromosome 2AS (Supplementary Table 7) were used as fixed effects and they included the STS markers (*cslVrgal3, IWB11136, Ventriup, WGGB156*, and *WGGB159*) and the GBS marker, 2A_14418709 (14,418,709 bps and 8.9 cM). Similar to the diversity and breeding panels, the highest mean prediction accuracies with the different models in the full-sibs panel was observed in the blast BLUEs dataset (0.65–0.72), that were 29.2–36.8% higher than the mean prediction accuracies of the individual environments. When the mean prediction accuracies of the environments across all the models were considered, we observed that it was the highest in Okinawa 2019 FP (0.68 ± 0.04) dataset and lowest in Jashore 2019 FP (0.41 ± 0.03) dataset.

The two-tailed *t*-tests for the significance of the mean differences between the prediction accuracies obtained from different models indicated that they were not significant at a threshold of 0.005 for the GBLUP + fixed effects and fixed effects models (Mean difference = 0.005, *p-*value = 0.19). We also observed that the fixed effects, Bayes B and GBLUP + fixed effects models had significantly higher prediction accuracies compared to the GBLUP model, with the mean differences ranging between 0.05 and 0.08 and the *p-*values for the test of significance of the mean differences ranging between 3.2 × 10^–7^ and 2.9 × 10^–8^. Similarly, the prediction accuracies from the GBLUP + fixed effects and the fixed effects models were significantly higher than those from the Bayes B model with a mean difference of 0.03 and the *p-*value for the test of significance of the mean differences ranging between 1.9 × 10^–4^ and 1.1 × 10^–5^.

#### Phenotypic Selection vs. Estimated Breeding Value Based Selection for Blast in the Full-Sibs Panel

**FIGURE 8:**
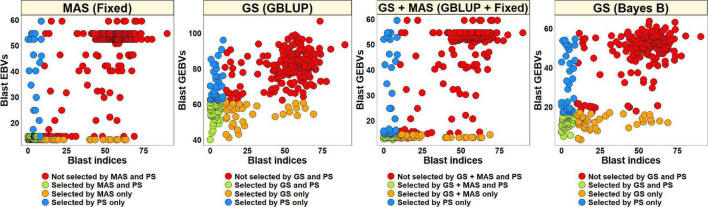
Comparison of phenotypic selection (PS) of the best linear unbiased estimates of blast indices across environments with: (i) marker assisted selection (MAS) using the estimated breeding values (EBVs) obtained from the fixed effects model (fixed) (ii) genomic selection (GS) using the genomic estimated breeding values (GEBVs) obtained from the genomic best-linear unbiased prediction (GBLUP) and Bayes B models and (iii) GS + MAS using the GEBVs obtained from the GBLUP and fixed effects (GBLUP + Fixed) model in the Caninde#1 × Alondra full-sibs panel comprising 298 lines.

The blast BLUEs dataset was used to select 82 lines (27.5%) with BLUEs less than 10 and a similar number of lines were selected from the EBVs obtained from different models (Figure 8). We observed that MAS based on EBVs from the fixed effects model had the highest percentage of overlap with PS, resulting in 75.6% of the lines selected by both and 90.7% of the lines discarded by both methods. The GEBVs obtained from the GBLUP + fixed effects, Bayes B and GBLUP models resulted in selection of 59.8, 57.3, and 58.5% lines, respectively, that were also selected by PS and discarding of 84.7, 83.8, and 84.3% lines, respectively, that were also discarded by PS.

#### Blast Distribution and Prediction Accuracies in Subsets of Lines With and Without the 2NS Translocation in the Full-Sibs Panel

**FIGURE 9:**
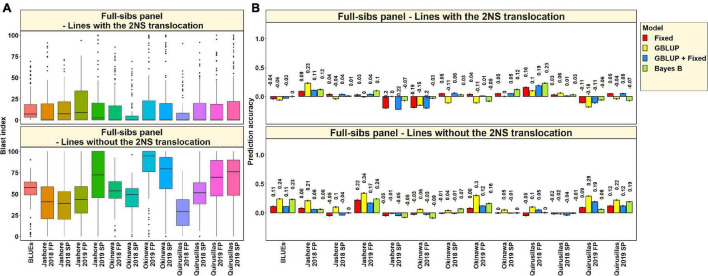
**(A)** Boxplots showing the wheat blast indices in 117 lines with the 2NS translocation in the full-sibs panel and 144 lines without the 2NS translocation in the full-sibs panel. **(B)** Twofold cross validation prediction accuracies for blast response in 117 lines with the 2NS translocation and 144 lines without the 2NS translocation in the full-sibs panel using the fixed effects (Fixed), genomic best linear unbiased prediction (GBLUP), GBLUP and fixed effects (GBLUP + Fixed) and Bayes B models. In **(A,B)**, FP refers to the first planting, SP refers to the second planting and BLUEs refer to the best linear unbiased estimates of blast indices across the different environments.

In the 117 full-sibs with the 2NS translocation, we observed that the mean blast index ranged between 7.8 ± 16.1 and 19.5 ± 31.3 in the different environments (Figure 9A). In the 144 full-sibs without the 2NS translocation, the mean blast index ranged between 29 ± 20.8 and 82.9 ± 26.2 in the different environments. The mean prediction accuracies for blast across the different environments for the lines with the 2NS translocation in the full-sibs panel ranged between 0.03 ± 0.09 using the Bayes B model and −0.02 ± 0.11 using the GBLUP model (Figure 9B). Similarly, for the lines without the 2NS translocation in the full-sibs panel, the mean prediction accuracies ranged between 0.15 ± 0.12 using the GBLUP model and 0.04 ± 0.08 using the fixed effects model. The markers used as fixed effects in the different datasets for the lines with and without the 2NS translocation in the full-sibs panel are given in Supplementary Tables 8, 9, respectively.

We observed that the mean prediction accuracy across all the environments and models was significantly higher in the subset of lines without the 2NS translocation compared to the subset of lines with the 2NS translocation (mean difference = 0.07, *p-*value = 5.7 × 10^–4^). In the full-sibs panel lines with the 2NS translocation, we observed that Quirusillas 2018 FP had the highest mean prediction accuracy (0.17 ± 0.05) across all the models, and Okinawa 2018 FP had the lowest mean prediction accuracy (−0.14 ± 0.08). In full-sib panel lines without the 2NS translocation, the mean prediction accuracy across all the models was the highest in Jashore 2019 FP (0.24 ± 0.07) and the lowest in Jashore 2019 SP (−0.05 ± 0.03).

## Discussion

We have successfully evaluated genomic prediction for wheat blast in three panels using the GBLUP and Bayes B models and compared the genomic prediction accuracies with those from the fixed effects, GBLUP + fixed effects and ABLUP models, to understand the relative advantage of using genome-wide markers. On average, across all the panels and environments analyzed in this study, the GBLUP + fixed effects model (0.63 ± 0.13) and the fixed effects model (0.62 ± 0.13) were the best models for predicting blast, followed by the Bayes B (0.59 ± 0.11), GBLUP (0.55 ± 0.1), and ABLUP (0.48 ± 0.06) models. Our results also indicated that there was no significant difference in the prediction accuracies from the GBLUP and Bayes B genomic prediction models in the diversity panel, as also observed in previous studies (Heslot et al., 2012; Juliana et al., 2017b). However, in the other two panels, the Bayes B model gave significantly higher accuracies compared to the GBLUP model, probably because the Bayes B model assumptions fitted well the genetic architecture of blast response in these panels, where the 2NS translocation had a large effect.

On comparing blast prediction accuracies from the genomic prediction models (GBLUP and Bayes B) with prediction accuracies from the fixed effects model and the GBLUP + fixed effects model, we observed: (i) no significant differences between the fixed effects, GBLUP + fixed effects and the Bayes B models in both the diversity and breeding panels and (ii) significantly higher prediction accuracies from the fixed effects model and GBLUP + fixed effects model compared to the genomic prediction models in the full-sibs panel. These results are contrasting to previous studies that have reported the superiority of genomic prediction models over the fixed effects model for some diseases in wheat (Rutkoski et al., 2012; Juliana et al., 2017b) and higher accuracies by integrating genomic prediction and the fixed effects model (Odilbekov et al., 2019; Sehgal et al., 2020). However, given that blast response in all the panels in this study was predominantly controlled by the 2NS translocation (He et al., 2020, 2021; Juliana et al., 2020a), our results are in agreement with Juliana et al. (2017a), who reported that for seedling leaf and stripe rust resistance, where a single gene had a large effect on the disease response in the population, the fixed effects model and the GBLUP + fixed effects model perform similar to or slightly better than the genomic prediction models. Hence, our findings have important implications for wheat blast predictions in environments where the resistance is determined by the 2NS translocation and indicate that in such environments, a fixed effects model with one to few markers tagging the 2NS translocation would be sufficient and genome-wide markers may not lead to a significant increase in blast prediction accuracies.

The 2NS translocation linked markers that were effective in predicting blast response in more than a fold or dataset in this study included the Illumina Infinium 15K BeadChip markers, Kukri_c22599_114 and Tdurum_contig29983_490 (El Hanafi et al., 2021); GBS markers, 2A_1686041, 2A_1872142, 2A_718152 and 2A_14418709 and STS markers, *cslVrgal3, IWB11136, Ventriup, WGGB156*, and *WGGB159*, all of which can be used to select for the 2NS translocation based blast resistance. But, it should also be noted that the 2NS translocation-based blast resistance is incomplete and sometimes background-dependent (Cruz et al., 2016b; Cruppe, 2020), with reports of the MoT isolates in Brazil (Ceresini et al., 2018) and Paraguay (Singh et al., 2016) having overcome the 2NS translocation-based blast resistance and hence relying on only one large effect resistance locus is not recommended, as it could result in selection pressure on the MoT populations (Cruz and Valent, 2017; Cruppe et al., 2020). However, in such cases where there is a risk of resistance breakdown and narrowing down the genetic variation for blast resistance by using predictions based on only one locus, the 2NS translocation-based markers can still be used to predict and select against the translocation.

Comparison of genomic and pedigree-based prediction accuracies indicated that in both the diversity and breeding panels, the ABLUP model resulted in the lowest prediction accuracies. This is consistent with previous studies that have reported superiority of genomic prediction over prediction prediction (Crossa et al., 2010; Spindel et al., 2015), while other studies have also reported similar accuracies from both (Juliana et al., 2017a,b, 2018, 2020b). However, we also observed that the ABLUP blast prediction accuracies were 85.4 and 83.6% of the mean genomic prediction accuracies from the Bayes B model in the different datasets of the diversity panel and breeding panel, respectively. This implies that although pedigree-based prediction for blast does not result in the highest accuracy, pedigree relationships can also be useful in predicting blast resistance, when genotyping data is not available or affordable.

Among the three sites of blast evaluation and prediction in this study, our results showed that Okinawa (0.63 ± 0.09) had the highest mean prediction accuracy across the different panels, models, years and planting times, followed by Quirusillas (0.59 ± 0.1) and Jashore (0.45 ± 0.06). Our results also indicated that the blast BLUEs dataset was the best predicted in all the three panels and accuracies in the BLUEs datasets were 4.7–45.5% higher than the mean prediction accuracies observed in the individual environments. One possible explanation to this is that the BLUEs obtained from multi-environment evaluations are most likely to be close to the true breeding values of the genotypes and hence predicted with the highest accuracy, thereby making them more robust for utilization in predictive breeding, compared to single-environment phenotypic observations. Another interesting observation in our study was that across all the environments, panels and models, the prediction accuracies from the two planting times were not significantly different (FP mean prediction accuracy: 0.56 ± 0.12; SP mean prediction accuracy: 0.57 ± 0.11), indicating that highly correlated blast indices in different planting times result in similar prediction accuracies.

Among the three panels evaluated for wheat blast prediction, the breeding panel had the highest mean prediction accuracy (0.66 ± 0.1), followed by the diversity panel (0.59 ± 0.13) and full-sibs panel (0.54 ± 0.1). This is a promising outcome of this study indicating that blast can be predicted with moderate to high predictabilities in all these panels, and hence prediction-based selection for wheat blast can be successfully implemented in any historic germplasm, breeding lines and sister lines. However, we could not directly compare prediction accuracies across panels, because of the different sizes of the panels, the different genotyping platforms used and also the different blast distributions in these panels. For example, the breeding panel had the highest number of resistant lines (48–62.5%) with a blast index of zero and this might have also contributed to high prediction accuracies.

This study is also unique because three different whole-genome marker platforms, the Illumina Infinium 15K BeadChip, GBS and DArTseq were evaluated for predicting wheat blast. Considering only the two genomic prediction models (GBLUP and Bayes B), we observed that the breeding panel genotyped using GBS was the best predicted (0.66 ± 0.07), followed by the diversity panel genotyped using the Illumina Infinium 15K BeadChip (0.60 ± 0.09) and the full-sibs panel genotyped using the DArTseq platform (0.51 ± 0.1). While previous studies have reported the superiority of GBS over both the DArTseq (Juliana et al., 2017b) and array-based platforms (Elbasyoni et al., 2018), the differences in prediction accuracies using these three platforms cannot be compared *per se* in this study, because of the aforementioned reasons (different panel sizes and blast distributions across panels) and none of the panels were genotyped using all the platforms. Hence, further studies on genomic predictions in different panels genotyped using the same genotyping platform are essential to compare blast predictabilities across different panels. Using a common platform to genotype different panels would also be useful to explore beyond the cross-validation strategy evaluated in this study and evaluate genomic prediction for blast across panels to understand how well one panel can be predicted from another. This would be akin to a practical GS implementation scenario, where breeders would be interested in predicting the blast response of lines from new panels using any existing panel. Since genomic prediction accuracies be lower in across-panel predictions compared to within-panel predictions (Juliana et al., 2019), it is important to evaluate across-panel predictions for wheat blast.

This study was also aimed to test the hypothesis that GS would perform better than MAS and pedigree-based selection for wheat blast. On average, across all the datasets and panels, MAS led to the selection of the highest percentage (88.5%) of lines selected by PS and discard of the highest percentage of lines (91.8%) that were discarded by PS. In contrast, on average, GS GBLUP and GS Bayes B only led to the selection of 75.2 and 80.6% of the lines that were selected by PS and discard of 81.9 and 87.4% of the lines that were discarded by PS, respectively. These results clearly indicated that MAS outperformed GS in our study, despite the phenotypic responses being continuous and indicating quantitative genetic control. However, pedigree-based selection, on average led to the selection of 77.3% of the lines that were selected by PS and the discard of 71.9% of lines that were discarded by PS and hence GS was superior to pedigree-based selection as hypothesized. It is also interesting that in a previous study comparing GS and PS for grain yield which is a highly quantitative trait, GS could select a maximum of 70.9% of the top lines and discard 71.5% of the poor lines (Juliana et al., 2018) at a selection intensity of 0.5, which is significantly lower than the percentage overlap with PS in this study, owing to less complex genetic architecture of wheat blast resistance in the panels used in this study.

We compared the prediction accuracies from different models obtained from subsets of lines with and without the 2NS translocation and the mean prediction accuracies across the different panels were 0.03 ± 0.16 (ranged between −0.22 and 0.45) and 0.16 ± 0.18 (ranged between −0.09 and 0.57), respectively. While the mean prediction accuracies in the subsets of lines were significantly lower than the mean prediction accuracies obtained in the full set of lines in each panel, our results demonstrate the possibility of implementing GS for blast in panels of lines without the 2NS translocation. However, it should be noted that our observations of blast predictions in lines with and without the 2NS translocation were done using subsets of few lines (53, 185, and 117 lines from the three panels had the 2NS translocation and 119, 47, and 144 lines from the three panels that did not have the 2NS translocation), and hence larger panels are needed to further understand the prediction accuracies for blast in panels of lines with and without the 2NS translocation. The higher blast predictabilities in the subsets of lines without the 2NS translocation could be because of the low variability in the blast indices in the lines with the 2NS translocation (mean blast indices ranged between 1.5 and 19.5) and the moderate to high variability in the blast indices (mean blast indices ranged between 14.3 and 82.9) in subsets without the 2NS translocation. We also observed that the mean prediction accuracies using the fixed effects model were very low (less than 0.10 in most subsets except in the lines without the 2NS translocation in the diversity panel), and the markers that were used in the different folds and datasets of the fixed effects model were inconsistent, indicating that the fixed effects model is not an ideal choice when there are no large effect consistent markers associated with blast in the panels.

Overall, this study has provided important insights into the genomic predictability of wheat blast and the prospects of implementing GS and MAS for the disease. One caveat in this study is that in all the three panels, blast resistance was controlled to a large extent by the 2NS translocation and hence further studies on genomic prediction of quantitative blast resistance in panels where resistance is not controlled by the 2NS translocation is needed. In conclusion, we have demonstrated that in populations where blast resistance is controlled by the 2NS translocation, MAS using few markers tagging the 2NS translocation can be used for accelerating predictive breeding for blast. This is a key finding of this study that opens several opportunities for wheat breeding programs to:

(i) Screen a subset of lines in the blast hot-spots and use that phenotyping data to predict the blast breeding values for other related lines, as demonstrated in this study where we evaluated genomic prediction assuming that a half of the lines were phenotyped.
(ii) Use the predicted breeding values to complement selection based on the phenotype and increase the selection accuracy.
(iii) Use the 2NS translocation-associated molecular markers to select for or against the 2NS based-blast resistance without phenotyping.
(iv) Scale-up selection for blast resistance to early generations of the breeding program that have been genotyped, but are in large numbers to be phenotyped. For example, the CIMMYT global wheat program screens international nurseries (200-300 lines) derived from the stage 3 yield trials for blast resistance, but about 9,000 stage 1 yield trial lines are genotyped each year. Here, the international nurseries can be used as training populations to predict the blast breeding values of the large set of stage 1 yield trial lines, thereby saving substantial cost and resources. In this case, GS can provide an advantage over MAS, as the same genotyping data can be used to select for multiple traits in the early generations.
(v) Sparse-test genotypes in different blast hot-spots in which not all the genotypes are grown in all the environments (Jarquin et al., 2020). For example: when there are cost-constraints for breeding programs to evaluate blast in multiple sites, then sparse-testing can be implemented in correlated sites.
(vi) For non-2NS resistance based predictive breeding, since screening a large number of lines for blast in field conditions to build training sets is challenging, greenhouse testing of blast can be used to primarily identify new resistance genes. This can be followed by obtaining GEBVs of the selected lines and then the best lines using PS and GS can be advanced for multilocation testing. Simultaneous selection against the 2NS translocation can also be performed using molecular markers, to facilitate the identification of non-2NS based resistance.

## Conclusion

In conclusion, while this study demonstrates the potential of MAS and GS for wheat blast resistance breeding, we would also like to emphasize that continued efforts to use genomic tools to identify non-2NS based sources of blast resistance in wheat is critical, which will involve coordinated high-throughput genomics and phenomics approaches.

## Data Availability Statement

The original contributions presented in the study are included in the article/Supplementary Material, further inquiries can be directed to the corresponding author/s.

## Author Contributions

PJ performed the analyses and drafted the first draft of the manuscript. PS and RS designed the study and supervised the analysis. XH, RI, BA, and FM were involved in blast phenotyping. JP and SS were involved in generating the genotyping data. GS, AC, PS, RS, and AJ provided germplasm and funds. All authors reviewed and approved the final version of the manuscript.

## Conflict of Interest

The authors declare that the research was conducted in the absence of any commercial or financial relationships that could be construed as a potential conflict of interest.

## Publisher’s Note

All claims expressed in this article are solely those of the authors and do not necessarily represent those of their affiliated organizations, or those of the publisher, the editors and the reviewers. Any product that may be evaluated in this article, or claim that may be made by its manufacturer, is not guaranteed or endorsed by the publisher.

## Footnotes

1
www.jmp.com

## Supplementary Material

Supplementary Data 1 Wheat blast indices of the lines in the diversity, breeding and full-sibs panel that were evaluated in Jashore, Okinawa and Quirusillas in two different planting dates indicated as first planting (FP) and second planting (SP) and the datasets are referred to by the site followed by the harvest year and planting time.
