# Supplementary material for: Genomic Selection for Wheat Blast in a Diversity Panel, Breeding Panel and Full-Sibs Panel
Source: Front Plant Sci. 2022 Jan 7;12:745379. doi: 10.3389/fpls.2021.745379 (PMC8782147; doi:10.3389/fpls.2021.745379)
Supplement: Supplementary file 2 [file Data_Sheet_2.docx]

**Supplementary Table 1: Markers used as fixed effects in the two folds of the fixed effects model in the diversity panel with 172 diverse spring wheat genotypes**

| Dataset | Fold 1 | Fold 2 |
| --- | --- | --- |
| Jashore 2018 FP | AX-95195576, Excalibur_c63009_102, BS00065873_51, AX-94796636, BS00041063_51 | BS00108733_51, RFL_Contig5022_2001 |
| Jashore 2018 SP | wsnp_Ex_c32905_41484291, Excalibur_c32467_676 | RAC875_c19904_308,  IACX2540, AX-94684111, Excalibur_c92705_94, Kukri_c35146_2094 |
| Okinawa 2018 FP | AX-94629608 | Kukri_c22599_114 |
| Okinawa 2018 SP | *Ventriup* | Tdurum_contig29983_490 |
| Quirusillas 2018 FP | Tdurum_contig29983_490 | Kukri_c22599_114 |
| Quirusillas 2018 SP | Tdurum_contig11802_864 | AX-95215927, Kukri_c31776_1621 |
| Quirusillas 2019 FP | Tdurum_contig29983_490 | wsnp_Ku_c33374_42877546 |
| Quirusillas 2019 SP | Kukri_c22599_114 | Kukri_c22599_114 |
| BLUEs | Tdurum_contig29983_490, Kukri_c22599_114 | Tdurum_contig29983_490 |

FP – First planting, SP – Second planting, BLUEs – Best linear unbiased estimates

**Supplementary Table 2: Markers used as fixed effects in the two folds of the fixed effects model in 53 lines with the 2NS translocation from the diversity panel**

| Dataset | Fold 1 | Fold 2 |
| --- | --- | --- |
| Jashore 2018 FP | Kukri_c25451_119, AX-94891670,  AX-94566622 | AX-95177447, JD_c3358_1128 |
| Jashore 2018 SP | NA | NA |
| Okinawa 2018 FP | NA | NA |
| Okinawa 2018 SP | AX-94509097 | wsnp_Ku_c8391_14261321,  AX-95220729 |
| Quirusillas 2018 FP | AX-94454402, Tdurum_contig96049_200 | CAP12_c8163_118 |
| Quirusillas 2018 SP | TA001999-1182 | Tdurum_contig13879_352,  AX-94702227 |
| Quirusillas 2019 FP | NA | NA |
| Quirusillas 2019 SP | BS00110130_51 | Ra_c21676_178 |
| BLUEs | AX-94870458,  tplb0033f11_1381 | AX-95115363, IAAV5917 |

FP – First planting, SP – Second planting, BLUEs – Best linear unbiased estimates

**Supplementary Table 3: Markers used as fixed effects in the two folds of the fixed effects model in 119 lines without the 2NS translocation from the diversity panel**

| Dataset | Fold 1 | Fold 2 |
| --- | --- | --- |
| Jashore 2018 FP | wsnp_Ex_rep_c67543_66165372,  AX-94629338, IACX3991 | BS00110452_51, Tdurum_contig14130_315, AX-94769850 |
| Jashore 2018 SP | AX-94652755, AX-94866977 | wsnp_Ex_rep_c68504_67334656, wsnp_Ex_c7168_12311649, AX-94769850, RAC875_rep_c72023_267 |
| Okinawa 2018 FP | BS00049548_51, AX-94703603, Tdurum_contig51422_127,  AX-95253498 | D_contig17313_245, Kukri_rep_c110911_477, BS00082084_51 |
| Okinawa 2018 SP | AX-94636903 | Tdurum_contig58312_119, Kukri_c18055_1740, wsnp_Ex_c23598_32826926, Tdurum_contig83837_251 |
| Quirusillas 2018 FP | RFL_Contig4186_914 | BS00110940_51, wsnp_Ex_c11229_18163892 |
| Quirusillas 2018 SP | BS00110940_51, wsnp_Ex_c11229_18163892 | AX-94892480, AX-95096194, wsnp_Ex_rep_c72011_70562321 |
| Quirusillas 2019 FP | Kukri_c10377_112 | wsnp_Ex_c7196_12357989, tplb0025i05_1836, GENE.4086_115, Tdurum_contig76709_195 |
| Quirusillas 2019 SP | wsnp_Ex_rep_c68194_66973531 | AX-94466287, IAAV6464, BS00062715_51 |
| BLUEs | AX-94392216, AX-95096194 | BobWhite_c47144_153, AX-95215927, AX-95096194, wsnp_Ku_c35386_44598937 |

FP – First planting, SP – Second planting, BLUEs – Best linear unbiased estimates

**Supplementary Table 4: Markers used as fixed effects in the two folds of the fixed effects model in the breeding panel with 248 lines**

| Dataset | Fold 1 | Fold 2 |
| --- | --- | --- |
| Okinawa 2018 SP | 2A_718152, 2A_1686041, 2A_1872142 | 2A_718152, 2A_1686041, 2A_1872142, 2A_2367215 |
| Quirusillas 2018 FP | 2A_718152 | 2A_718152 |
| Quirusillas 2019 FP | 2A_718152 | 2A_718152, 2A_1686041, 2A_1872142 |
| Quirusillas 2019 SP | 2A_718152, 2A_1872142 | 2A_718152, 2A_1686041, 2A_1872142 |
| BLUEs | 2A_718152 | 2A_718152 |

FP – First planting, SP – Second planting, BLUEs – Best linear unbiased estimates

**Supplementary Table 5: Markers used as fixed effects in the two folds of the fixed effects model in 185 lines with the 2NS translocation from the breeding panel**

| Dataset | Fold 1 | Fold 2 |
| --- | --- | --- |
| Okinawa 2018 SP | 5A_559517035, 2B_752475513, 4B_20355661 | 1D_141006535, 7B_700903488 |
| Quirusillas 2018 FP | 5A_559517035, 2B_752475513, 4B_20355661 | 1D_141006535, 7B_700903488 |
| Quirusillas 2019 FP | UN_35479688, 6D_18671500, 7A_59616137 | 7D_29949503, UN_296101295, 2A_271909782 |
| Quirusillas 2019 SP | 6A_76926356 | 3D_40548616, 4D_336833335, 7B_116377571, 7A_13179057 |
| BLUEs | 3B_17109541, 3B_740133519, 6A_76926356, 1A_571348798 | 6D_458009032, 3D_40548616 |

FP – First planting, SP – Second planting, BLUEs – Best linear unbiased estimates

**Supplementary Table 6: Markers used as fixed effects in the two folds of the fixed effects model in 47 lines without the 2NS translocation from the breeding panel**

| Dataset | Fold 1 | Fold 2 |
| --- | --- | --- |
| Okinawa 2018 SP | 4A_738742632 | 5B_557138172, 4A_4593612 |
| Quirusillas 2018 FP | 2A_31045480, 7A_693692752, 7A_66984189 | 2B_50715982 |
| Quirusillas 2019 FP | 1B_601377919., 7B_704641603 | 3A_23856026 |
| Quirusillas 2019 SP | 5B_18066511 | 3A_699574461 |
| BLUEs | 6A_545127388, 4B_622455 | 7B_711427912 |

FP – First planting, SP – Second planting, BLUEs – Best linear unbiased estimates

**Supplementary Table 7: Markers used as fixed effects in the two folds of the fixed effects model in the full-sibs panel with 298 full-sibs**

| Dataset | Fold 1 | Fold 2 |
| --- | --- | --- |
| Quirusillas 2018 FP | *IWB11136* | *cslVrgal3* |
| Quirusillas 2018 SP | *WGGB156* | *WGGB159, cslVrgal3* |
| Quirusillas 2019 FP | *WGGB156, IWB11136, WGGB159* | *cslVrgal3, IWB11136, Ventriup* |
| Quirusillas 2019 SP | *WGGB156, cslVrgal3* | *WGGB156, cslVrgal3* |
| Jashore 2018 FP | *IWB11136* | *IWB11136* |
| Jashore 2018 SP | *WGGB156* | *WGGB156* |
| Jashore 2019 FP | *Ventriup* | *WGGB156* |
| Jashore 2019 SP | *WGGB156* | *IWB11136, WGGB156* |
| Okinawa 2018 FP | *WGGB156* | *IWB11136, WGGB156* |
| Okinawa 2018 SP | *WGGB156* | *IWB11136, WGGB156* |
| Okinawa 2019 FP | *cslVrgal3, IWB11136, WGGB156* | 2A_14418709*, cslVrgal3, IWB11136* |
| Okinawa 2019 SP | *WGGB156* | *cslVrgal3, IWB11136, WGGB156* |
| BLUEs | *cslVrgal3, IWB11136, WGGB156* | 2A_14418709*, cslVrgal3, IWB11136* |

FP – First planting, SP – Second planting, BLUEs – Best linear unbiased estimates

**Supplementary Table 8: Markers used as fixed effects in the two folds of the fixed effects model in 117 lines with the 2NS translocation from the full-sibs panel**

| Dataset | Fold 1 | Fold 2 |
| --- | --- | --- |
| Quirusillas 2018 FP | 7A_54762401, 2B_575904255, 6B_597558242, 6A_37451270, 7A_640164740 | UN_31931208, 5A_18653351, 7A_732326458 |
| Quirusillas 2018 SP | 1A_12130324, 2B_528839631 | UN_31931208, 2B_140852955, 2B_117988168, 2B_122693763, 6A_614373377 |
| Quirusillas 2019 FP | 3A_667351667, 7B_71240408, 7B_27599447, 2B_775343394, 2B_762891276 | 5A_502072031 |
| Quirusillas 2019 SP | 3A_667351667, 7B_71240408, 7B_27599447, 2B_775343394, 2B_762891276, 7B_46577932, 6B_72379426 | 5A_502072031 |
| Jashore 2018 FP | 4B_619075571, 2A_728790463, 2A_753013019, 4B_25886167 | 6A_44973108, 5A_503430449 |
| Jashore 2018 SP | 6A_603138042, UN_288925099 | 3B_825868283, 3B_811421481 3B_716339022 |
| Jashore 2019 FP | 7D_618712652, 7B_113767734 | 4D_3356525, 5A_577795502, 5D_424046570 |
| Jashore 2019 SP | 1B_542641861, 2B_682193316 2B_528839631 | 6B_107070800, 4B_548121036 |
| Okinawa 2018 FP | 2B_762891276, 7B_516790926, UN_91646358, 6A_22186473, 7B_526239910, 7B_532270352, 7B_647757237, 3B_752317171 | 7B_749571927, 3B_825868283, 7B_616469435, 7A_679954508 |
| Okinawa 2018 SP | 6A_13815254, 3A_667351667, 1A_30307262 | 2A_34350090, 3A_626098128, 7A_540872088, 3B_825868283, 7A_679954508, 7A_566094708, UN_36755909, 6B_701146766 |
| Okinawa 2019 FP | 6A_22186473, 2B_784905752, 2B_777653055, 2B_733077296 | 7B_749571927, 6B_700666990, 7B_526239910, 6A_583271131 |
| Okinawa 2019 SP | 6A_22186473, 2D_580241467, 7B_647757237 | 7B_749571927, 7B_602304901, UN_31931208, 3B_716339022 |
| BLUEs | 2B_784905752, 2B_682193316 | 7B_749571927, 3B_716339022, 7B_602304901 |

FP – First planting, SP – Second planting, BLUEs – Best linear unbiased estimates

**Supplementary Table 9: Markers used as fixed effects in the two folds of the fixed effects model in 144 lines without the 2NS translocation from the full-sibs panel**

| Dataset | Fold 1 | Fold 2 |
| --- | --- | --- |
| Quirusillas 2018 FP | 7B_48046898, 1B_30218847, 1A_532766627 | 7A_566060534, 4A_596830631, 4A_605005072, 2B_195530321 |
| Quirusillas 2018 SP | 1B_30218847, 2B_748152909, 4A_114487333 | 6B_72379426, 1B_119579900 |
| Quirusillas 2019 FP | 1D_37887967, 5A_37970873, 1D_52680610, 2B_769950936 | 6B_27697090, 4B_21573479, 1B_653794821, 7A_38241544, 7A_675041656 |
| Quirusillas 2019 SP | 1B_299551612, 3B_810657303, 6D_2074705, 6A_23587868, 2B_748152909 | 6B_27697090, 6B_664457926 |
| Jashore 2018 FP | 4A_631976748, 3B_812610831 | 4B_21573479, 2B_775486141, 2A_14418709, 7B_750602542, 7B_63769728 |
| Jashore 2018 SP | 4B_4216511, 1B_615614208, 7B_742273727, 5A_514092701, 6B_5829982 | 2D_16587494, 7A_44352216, 6B_641961305 |
| Jashore 2019 FP | 3B_725118688, 3B_711641981, 1A_4206794, 5B_71300457, 1A_7214008 | 4A_629486754, 3A_20238715, 2B_197708090 |
| Jashore 2019 SP | 6D_1489368, 5B_681727782, 2B_22307595, 3B_716302405, 4B_4216511, 4B_25886167, 5A_548759453, 5A_15724613, 1D_52602336, 5A_9593480 | 3A_492040011, 3A_492337420, 7B_113767734, 6A_23364183, 5D_439972803, 1D_34010984, 7A_48831502, 4A_28598225 |
| Okinawa 2018 FP | 2B_682193316, 2B_775486141, 5B_577229443, UN_16472260, 7A_8291551 | 2D_57265325, 4A_612532237, 5D_543229204, 5A_52711229 |
| Okinawa 2018 SP | 2B_682193316, 3A_97477038, 6D_25614526, 1D_52602336, 6B_600787266, 1B_545163522 | UN_162967131 |
| Okinawa 2019 FP | 7A_8291551, 2B_682193316, 3A_621592828, 6D_25614526, UN_94045128, 2B_400995661 | UN_162967131, 5A_69140651, 1B_619588077 |
| Okinawa 2019 SP | 6A_24845173, 2B_682193316, 3A_734547664 | 1B_619588077, 2D_1664661 |
| BLUEs | 2B_682193316, 5B_577229443, 6D_25614526 | 1B_619588077, 3A_26299408, UN_162967131, 3B_810657303, 1D_52680610 |

FP – First planting, SP – Second planting, BLUEs – Best linear unbiased estimates
